# Studies on *Emblica officinalis* Derived Tannins for Their Immunostimulatory and Protective Activities against Coccidiosis in Industrial Broiler Chickens

**DOI:** 10.1155/2014/378473

**Published:** 2014-01-22

**Authors:** Qari Muhammad Kaleem, Masood Akhtar, Mian Muhammad Awais, Muhammad Saleem, Muddassar Zafar, Zafar Iqbal, Faqir Muhammad, Muhammad Irfan Anwar

**Affiliations:** ^1^Immunoparasitology Laboratory, Department of Parasitology, University of Agriculture, Faisalabad, Pakistan; ^2^Faculty of Veterinary Sciences, Bahauddin Zakariya University, Multan, Pakistan; ^3^Department of Pathobiology, Sub Campus Jhang, University of Veterinary and Animal Sciences, Lahore, Pakistan; ^4^Nuclear Institute for Agriculture and Biology, Faisalabad, Pakistan; ^5^Department of Biochemistry, PMAS-Arid Agriculture University, Rawalpindi, Pakistan; ^6^Department of Physiology and Pharmacology, University of Agriculture, Faisalabad, Pakistan; ^7^Poultry Research Institute, Office of Deputy District Livestock Officer (Poultry), Faisalabad, Pakistan

## Abstract

The present study reports the effect of *Emblica officinalis* (EO) derived tannins on humoral immune responses and their protective efficacy against *Eimeria* infection in chickens. Tannins were extracted from EO and characterized by HPLC. EO derived tannins (EOT) and commercial tannins (CT) were orally administered in broiler chicks in graded doses for three consecutive days, that is, 5th-7th days of age. On day 14 after administration of tannins, humoral immune response was detected against sheep red blood cells (SRBCs) by haemagglutination assay. Protective efficacy of tannins was measured against coccidial infection, induced by *Eimeria* species. Results revealed higher geomean titers against SRBCs in chickens administered with EOT as compared to those administered with CT and control group. Mean oocysts per gram of droppings were significantly lower (*P* < 0.05) in EOT administered chickens as compared to control group. Lesion scoring also showed the lowest caecal and intestinal lesion score of mild to moderate intensity in chickens administered with EOT. Further, significantly higher (*P* < 0.05) daily body weight gains and antibody titers were detected in EOT administered chickens as compared to those of CT administered and control groups. EOT showed the immunostimulatory properties in broilers and their administration in chickens boost the protective immunity against coccidiosis.

## 1. Introduction

*Emblica officinalis* (EO) belongs to family Euphorbiaceae and is commonly called amla [1]. It is generally present in almost all Asian countries including Pakistan [2]. All parts of this plant, particularly its fruit, are extensively used in Ayurvedic and Chinese herbal medicine [3]. The fruit of EO is a rich source of many bioactive molecules including alkaloids, carbohydrates, polyphenolics, essential amino acids, and vitamins especially vitamin C [4]. The higher concentration of vitamin C in amla makes it a strong antioxidant and antiaging agent [5–7]. It also contained tannic acid, ellagitannin, gallic acid, emblicanin A, emblicanin B, and ellagic acid along with flavonoids and kaempferol [2, 8, 9].

In traditional medicine, amla plant, its fruit, or its various constituents have been extensively used [3, 10] in different herbal formulations of Unani, Chinese, and Sidha systems of medicine to treat a variety of maladies [2, 10–12]. It favours longevity, improves digestion, reduces hyperthermia, normalizes blood parameters, alleviates asthmatic conditions, stimulates growth of hair, and strengthens heart and liver [13]. Moreover, its therapeutic activities in various eye ailments, dyspepsia, gastroenteritis, anemia, hyperglycemia, fatigue, and general weakness have also been reported [13–15]. Experimental studies revealed that *E. officinalis* possessed antimicrobial, antiviral and antifungal, hypolipidemic, antimutagenic, and immunomodulatory activities [2, 8].

*E. officinalis* contained high and low molecular weight polyphenolic compounds commonly called tannins, including pedunculagin, puniglucanin, emblicanin A, and emblicanin B [16, 17]. Tannins extracted from botanical origin reduced the size and incidence of skin tumor [18, 19], lung tumors [20], duodenal tumor [21], colonic tumor [22], and forestomach and pulmonary tumors [23] in mice. Its protective effects against esophageal, duodenal, pancreatic, hepatic, pulmonary, and mammary tumors had also been documented in animal models [24]. Some of the tannins, particularly acrimonium and ornithine B, potentiated the immune system by enhancing the activities of the natural killer cells (T cells) and macrophages [25, 26]. Keeping in view the diverse biological activities of amla and other plant derived tannins in different animal models, the present study was conducted to investigate the effects of *E. officinalis* derived tannins on the immune responses in chickens and their protective efficacy against avian coccidiosis.

## 2. Materials and Methods

### 2.1. Procurement and Processing of *Emblica officinalis*

Fresh fruits of EO were purchased from the local market of Faisalabad, Pakistan, and their authenticity was confirmed by the concerned botanist of the University of Agriculture, Faisalabad (UAF), Pakistan. The plant specimen was kept in the Ethno-veterinary Research and Development Centre, Department of Parasitology, UAF, as voucher No. 0176.

The fruits were washed with chlorinated water (chlorine 5–10 ppm) followed by distilled H_2_O, deseeded, chopped into small pieces, dried, and ground to powder form. The powder was sieved through a mesh (0.05 cm pore size) to get the optimum particle size of 0.5 mm [27]. The powder thus obtained was preserved under dry conditions at 4°C until further processing.

### 2.2. Elemental Analysis of *E. officinalis* Using Inductively Coupled Plasma-Optical Emission Spectrometer (ICP-OES)

The dried powder of *E. officinalis *was subjected to elemental analysis by using ICP-OES (OPTIMA 2100 DV; Dual View, Perkin Elmer, USA) to detect and quantify the heavy metals in the EO sample. Briefly, dried powder of *E. officinalis *(1 gm) was suspended in concentrated nitric acid (10 mL; Merck, Germany) in a 50 mL digestion flask. The flask was covered with watch glass and incubated overnight to subside the initial reaction. The suspension thus obtained was heated continuously for 12 hours until solid particles disappeared. After cooling, 72% perchloric acid (10 mL; Sigma-Adrich, USA) was added and heated gently followed by vigorous shaking to get a clear and colourless solution. The solution was cooled and 100 mL sample was transferred into a flask and allowed to stand undisturbed overnight followed by filtration (Whatman no. 1001-032). The filtrate thus obtained was used in elemental analysis. The conditions used in ICP analysis were as follows: nebulization gas flow rate: 0.80 L min^−1^; auxiliary gas flow rate: 0.2 L min^−1^; plasma gas flow rate: 15 L min^−1^; sample flow rate: 1.50 mL min^−1^; operating power: 1300 watt; view: axial and radial; interface: shear gas; sample uptake rate: 2.50 mL min^−1^; spray chamber: 1.0 mL min^−1^; nebuliser type: meinhard and nebuliser setup: instant. Further, the detection wavelengths (*λ*/nm) of different elements are shown in Table 1. The standards used in the analysis were prepared in the range of 0.05 to 10 mg/litre. The results were expressed as mean values of triplicate measurements.

### 2.3. Extraction of Tannins from EO

Tannins from the dried powder of *E. officinalis *were extracted following the method of Sánchez-Martín and his coworkers [28] with minor modifications. Briefly, powder (100 gm) was suspended in double distilled water (600 mL) followed by the addition of sodium hydroxide (5 gm; Merck, Germany). The mixture was subjected to continuous stirring at 90°C for one hour followed by centrifugation (1700 ×g for 40 minutes). Supernatant was collected and concentrated at 65°C in the water bath for 24 hours, and the resultant was used as tannin extract. The presence of tannins in the extract was confirmed by a colorimetric method [29].

### 2.4. HPLC Analysis of Tannins

The extracted tannins were analyzed on a Shimadzu-10A HPLC workstation (Japan) equipped with a quaternary gradient pump unit and UV/visible detector. The volume injection of extracted tannins and standards was 20 *μ*L, whereas isocratic distilled de-ionized water was used as mobile phase. The flow rate was 1 mL/minute. The analysis was performed at room temperature (26°C) on Shim-Pack CLC-ODS (C-18) column having 15 cm length, 4.6 mm internal diameter with 5 *μ*m particle size. Retention time and peak areas of standards of tannins were noted and calculated, respectively. These calculations of peak area from the respective chromatogram were employed for the estimation of tannic acid (tannins) concentration in *E. officinalis*.

### 2.5. Sterility and Safety Testing of *Emblica officinalis* Derived Tannins (EOT)

EOT were subjected to sterility testing using thioglycollate broth (Merck, Germany) to check the contamination, if any. Absence of growth in the broth confirmed the sterility of EOT. A preliminary pilot project was conducted to determine the safer dose limit of EOT in chickens. For this purpose, a total of forty-day-old industrial broiler chicks (Hubbard) were divided into 4 equal groups (*n* = 10). Three groups were administered orally with EOT at three different dose rates (0.75, 1.00, and 1.25 gm/kg body weight) for three consecutive days (5th, 6th, and 7th days of age), whereas control group (without EOT administration) was also raised for comparison with EOT-treated groups. The birds were monitored for seven days to observe the general behaviour of chickens along with feed and water intake. Findings revealed that chickens administered with EOT (1.25 gm/kg body weight) were dull, depressed with reduced feed and water intake. Moreover, the postmortem findings also showed pathological discolouration of liver, reactive bursa, and swollen kidneys in these chickens, whereas no such abnormalities were observed in other groups administered with EOT at a dose rate ≤1.00. Thus, administration of EOT at a dose rate of ≤1.00 gm/kg body weight was considered safe for their biological evaluation in broiler chickens.

### 2.6. Experimental Design

A total of 210 (1-day-old) broiler chicks (Hubbard) procured from local hatchery were kept in coccidia-free environment at the Experimental Station, Department of Parasitology, UAF. All the chicks were fed withdrawal feed and water *ad libitum*. Chickens in all the groups were vaccinated following the routine vaccination schedule [30]. Birds were acclimatized for 5 days and split into seven equal groups (*n* = 30), namely, A_1_, A_2_, A_3_, B_1_, B_2_, B_3_, and Control. Groups A_1_, A_2_, and A_3_ were administered with EOT, whereas B_1_, B_2_, and B_3_ were administered with commercial tannic acid as parallel positive control groups (CTA; Sigma-Aldrich, USA) at different dose rates for three consecutive days, that is, 5th, 6th, and 7th days of age according to the schedule as follows, whereas all the doses were constituted in 1 mL of phosphate buffered saline (PBS):

 A_1_: EOT at a dose rate of 0.50 gm/kg body weight;
 A_2_: EOT at a dose rate of 0.75 gm/kg body weight;
 A_3_: EOT at a dose rate of 1.00 gm/kg body weight;
 B_1_: CTA at a dose rate of 0.50 gm/kg body weight;
 B_2_: CTA at a dose rate of 0.75 gm/kg body weight;
 B_3_: CTA at a dose rate of 1.00 gm/kg body weight;
 C: PBS at a dose rate of 1.00 mL and served as negative control.

On day 14 after administration of different treatments, half of the chickens (*n* = 15) from each group were used for immunological evaluation and the remaining half for evaluation of protective efficacy against coccidiosis.

### 2.7. Infective Material

Sporulated oocysts of mixed species of genus *Eimeria* (local isolates), maintained in the Immunoparasitology Laboratory, UAF, were used in the present study. The infective dose was adjusted to 6.5 × 10^4^–7.0 × 10^4^ sporulated oocysts per 4 mL of PBS and mainly contained *E. acervulina, E. maxima, E. necatrix, *and *E. tenella*.

### 2.8. Immunological Evaluation

Sheep red blood cells (SRBCs) as nonpathogenic T-dependant antigens were used to demonstrate the antibody titer (Total Igs, IgM, and IgG) according to the methodology described by Qureshi and Havenstein [31]. In brief, on day 14 after administration of tannins, chickens were injected with SRBCs (5%) via intramuscular route (1 mL/chicken) followed by a booster after two weeks of primary injection. Blood was collected each at days 7 and 14 after primary and secondary injections and sera were separated from all the blood samples. Sera samples were evaluated for total immunoglobulins (Igs), IgM (mercaptoethanol-sensitive), and IgG (mercaptoethanol-resistant) anti-SRBCs antibodies by using microplate hemagglutination assay and results were expressed in terms of geomean titer (GMT).

### 2.9. Effect on the Development of Lymphoid Organs

Chickens from the experimental and control groups were individually weighed and slaughtered on day 42 of their age (last day of experiment). Lymphoid organs including bursa of fabricius, thymus, spleen, and caecal tonsils were incised out and weighed. The results were expressed in terms of lymphoid organ- live body weight ratios [32].

### 2.10. Evaluation of Protective Efficacy against Eimeria Infection

Chickens in all the groups (*n* = 15) were challenged with mixed species of *Eimeria* (local isolates) on day 14 after administration of tannins. All the groups were monitored for mortality [33], daily body weight gain, and oocysts per gram of droppings [34] from day 4 to day 12 after challenge. The caeca and intestine of the chickens that died during challenge experiment and those of survived/sacrificed at the end of the challenge experiment were monitored for lesion scoring [35]. Percent protection against lesions was also determined by using the formula described by Singh and Gill [36] as follows: [Average lesion score (IUG) − Average lesion score (IMG)/Average lesion score (IUG)] × 100, where IUG is the infected untreated group and IMG is the infected medicated group.

### 2.11. Assessment of Elevated Humoral Response against *Eimeria* Species by Enzyme Linked Immunosorbent Assay (ELISA)

Elevated humoral response in terms of antibody titres against *Eimeria* species used in the challenge experiment was determined by ELISA [37]. The optical density (OD) was read at 492 nm in an ELISA reader (BioTek-MQX200, USA). The mean absorbance values were recorded and the OD value was calculated. Positive and negative control sera were run in each plate and the corrected OD value was determined by using the formula as follows:

(1) ODcorrected=ODSample−ODNegative  control  of  plateODPositive  control  of  plate−ODNegative  control  of  plate.

### 2.12. Statistical Analysis

Two-way analysis of variance (ANOVA) and least significant difference (LSD) tests were used for the determination of statistical significance. The values of all the parameters analyzed statistically were considered significant at *P* < 0.05.

## 3. Results

### 3.1. Inductively Coupled Plasma (ICP) Analysis of EO Fruit

ICP analysis was performed to detect and quantify the minerals and heavy metals in the EQ fruit that was used for the extraction purpose. Results revealed the presence of both macro and microminerals in the dried EO fruit, whereas a heavy metal (nickel) was also detected in the dried powder of EO fruit but under permissible intake level. Other minerals and/or trace elements detected in the EO fruit were copper, magnesium, iron, manganese, and zinc (Table 1).

### 3.2. High Performance Liquid Chromatographic (HPLC) Analysis of EOT

**Figure 1:**
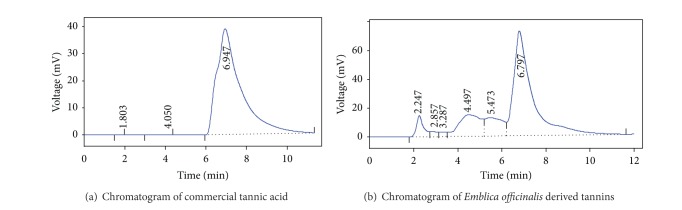
Chromatograms of *Emblica officinalis* derived and commercial tannins.

HPLC analysis of the EOT confirmed the presence of tannic acid in the extract when compared with the standard solution of tannic acid. Both EOT and standard solution of tannic acid showed the peaks in their respective chromatograms at a retention time of 6.947 which confirmed the presence of tannins in the extract (Figures 1(a) and 1(b)). Furthermore, the quantification of EOT sample by mathematical conversions showed that each gram of dried tannin extract contained 133.60 mg tannic acid and all the doses in the current experiment were calculated based upon this quantification.

### 3.3. Immunological Evaluation

Antibody titers detected by microplate haemagglutination assay revealed that oral administration of EOT and CTA resulted in higher total Igs, IgG, and IgM geomean titers (GMT) against SRBCs on days 7 and 14 after primary injection (PPI) of SRBCs as compared to control group. However, among the experimental groups administered with tannins, those who received EOT showed higher GMT values against SRBCs as compared to those who received CTA. With respect to dose response, in both EOT and CTA administered groups, the chickens administered with tannins (either EOT or CTA) at a dose rate of 1 gm/kg of body weight showed the maximum response in terms of the highest GMTs as compared to other groups administered with tannins either at a dose rate of 0.5 gm or 0.75 gm/kg of body weight. A similar response was detected on days 7 and 14 after secondary injection (PSI) of SRBCs (Table 2).

### 3.4. Effect on the Development of Lymphoid Organs

Effects of the oral administration of EOT and CTA on the development of lymphoid organs were calculated and results showed apparently higher percent organ-body weight ratios in EOT and CTA administered groups as compared to negative control administered with PBS, although the difference was statistically nonsignificant (*P* > 0.05) (data not shown).

### 3.5. Evaluation of Protective Efficacy against *Eimeria* Infection

Protective efficacy of EOT was determined in chickens of experimental groups in comparison with positive (CTA administered) and negative (PBS administered) control groups. Chickens of all the experimental and control groups were experimentally infected with mixed species of genus *Eimeria* (local isolates) on 14 day after administration of EOT and CT.

#### 3.5.1. Oocyst Count

**Figure 2:**
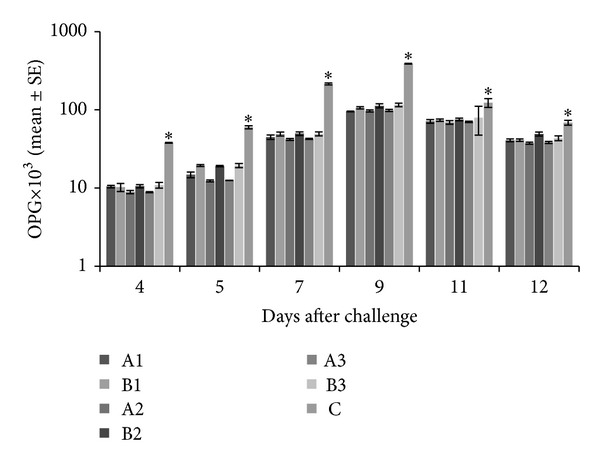
Oocysts per gram of droppings from day 4 to 12 after challenge in experimental and control chickens. A1 = *Emblica officinalis *derived tannins at 0.50 gm/Kg b. wt; B1 = commercial tannins at 0.50 gm/Kg b. wt; A2 =* Emblica officinalis *derived tannins at 0.75 gm/Kg b. wt; B2 = commercial tannins at 0.75 gm/Kg b. wt; A3 = *Emblica officinalis *derived tannins at 1.00 gm/Kg b. wt; B3 = commercial tannins at 1.00 gm/Kg b. wt; C = PBS-administered negative control (*significantly lower values).

The oocysts shed in droppings were counted from day 4 to day 12 after challenge with *Eimeria* species and results were expressed in terms of oocysts per gram of droppings (OPG) (mean ± SE). All the experimental groups administered with graded doses of tannins, either EOT or CTA, showed significantly lower (*P* < 0.05) OPG as compared to control, whereas among the experimental groups, the difference in OPG was statistically nonsignificant (*P* > 0.05) between the groups administered with tannins either EOT or CTA on a particular dose rate and day after challenge. The highest oocysts count (peak) was observed on day 9 after challenge in all the experimental and control groups (Figure 2).

#### 3.5.2. Daily Body Weight Gains

**Figure 3:**
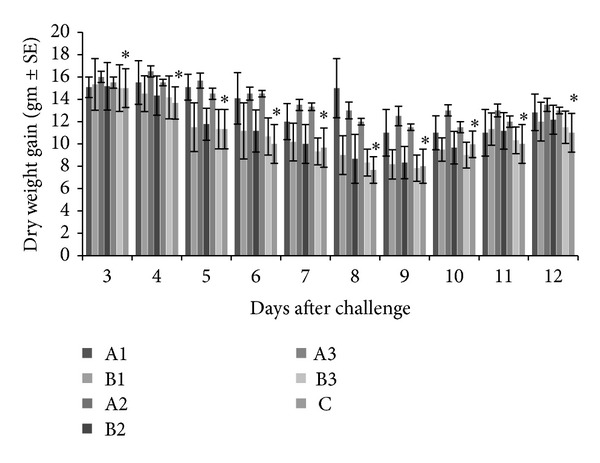
Daily weight gains from day 3 to 12 after challenge in experimental and control chickens. A1 = *Emblica officinalis *derived tannins at 0.50 gm/Kg b. wt; B1 = commercial tannins at 0.50 gm/Kg b. wt; A2 =* Emblica officinalis *derived tannins at 0.75 gm/Kg b. wt; B2 = commercial tannins at 0.75 gm/Kg b. wt; A3 = *Emblica officinalis *derived tannins at 1.00 gm/Kg b. wt; B3 = commercial tannins at 1.00 gm/Kg b. wt; C = PBS-administered negative control (*significantly lower values).

Daily weight gains were recorded from day 3 to day 12 after challenge and results showed that all the experimental groups treated with tannins, either EOT or CTA, had higher daily weight gains as compared to PBS-administered control group and the difference was statistically significant (*P* < 0.05). On the other hand, apparently higher daily weight gains were recorded in chickens administered with EOT as compared to those administered with CTA at similar dose rates, but the difference was statistically nonsignificant (*P* > 0.05) except on days 8 and 9 after challenge (Figure 3).

#### 3.5.3. Percent Protection

All the experimental and control groups were monitored for percent protection after challenge with *Eimeria* species. Results revealed maximum protection (60%) in group A_2_ administered with EOT (0.75 gm/kg of body weight) and minimal protection (26.67%) in PBS administered control group. On the whole, chickens administered with EOT showed the best response in terms of percent protection followed by those administered with CTA and PBS administered control groups, respectively (Table 3).

#### 3.5.4. Lesion Scoring and Percent Protection against Lesions

Chickens were scored for caecal and intestinal lesions after challenge using a scale of 0 to 4. In PBS administered control group, 93.33% of the chickens exhibited severe caecal lesions (3.0-4.0) and only 6.67% showed mild to moderate lesions (1.0-2.0). In tannins administered groups, the lowest caecal lesion score of severe intensity was developed in group A_3_ (53.33%), administered with EOT (1.0 gm/Kg body weight), whereas 73.33% of the chickens in each of group A_1_ and group A_2_ exhibited severe caecal lesions. On the other hand, a higher percent severe caecal lesions were observed in CTA administered chickens as compared to EOT administered ones. In case of intestine, chickens of all the experimental groups treated with tannins showed lower lesion scores as compared to the control group except group B_3_ (CTA; 1.0 gm/Kg body weight) which showed a higher percentage (86.67%) of severe lesion score as compared to control (80%). The lowest score (60%) of severe intestinal lesions (3.0-4.0) was recorded in group A_2_ (0.75 gm/Kg body weight) followed by groups A_1_, A_3_, and B_2_ each in which 66.67% of the chickens showed severe lesions on the intestine.

To access the protective efficacy of EOT against *Eimeria* induced lesions, percent protection against lesions was also calculated in all the groups and the highest protection (30%) against caecal lesions was recorded in chicken administered with EOT (1.00 gm/Kg body weight) and for intestinal lesions (25%) in those administered with EOT (0.75 gm/Kg body weight) (Table 3).

### 3.6. Antibody Responses to *Eimeria* Species

**Figure 4:**
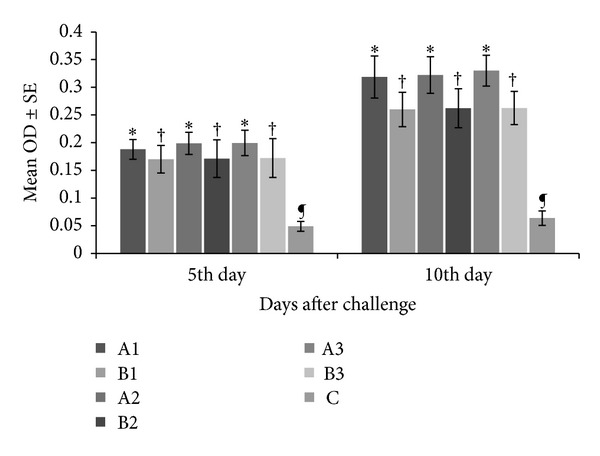
Serum antibody titers on day 5 and 10 after challenge with* Eimeria* species (local isolates). A1 = *Emblica officinalis *derived tannins at 0.50 gm/Kg b. wt; B1 = commercial tannins at 0.50 gm/Kg b. wt; A2 =* Emblica officinalis *derived tannins at 0.75 gm/Kg b. wt; B2 = commercial tannins at 0.75 gm/Kg b. wt; A3 = *Emblica officinalis *derived tannins at 1.00 gm/Kg b. wt; B3 = commercial tannins at 1.00 gm/Kg b. wt; C = PBS-administered negative control. Bars sharing similar letters on each particular day are statistically nonsignificant (*P* > 0.05).

The results of ELISA performed on sera samples obtained from experimental and control chickens are shown in Figure 4. On day 5 after challenge, chickens administered with EOT and CTA showed significantly higher (*P* < 0.05) mean absorbance values as compared to PBS-administered control group. On the other hand, among the tannins-treated groups, at a particular dose rate, chickens administered with EOT showed significantly higher (*P* < 0.05) OD values as compared to those administered with CTA. A similar trend was observed on day 10 after challenge; however, all the groups showed higher OD values on day 10 when compared with those on day 5 after challenge.

## 4. Discussion

Medicinal herbs have been traditionally used around the globe for centuries to modulate the immune activities and to cure various ailments in man and animals. Recent developments in scientific validation of herbs and their products for modern healthcare system have gained much momentum from the last two decades [38]. According to an estimate, more than eighty per cent of the modern research on drug discovery has been focused on botanical sources [39]. This drastic shift from synthetic agent to natural products may be due to more frequent adverse effects seen with the use of synthetics, which included development of drug resistance [40]. In this regard, EO is well known for its therapeutic activities and has been remained as an essential part of most of the herbal formulations [41]. EO is a rich source of different bioactive molecules including alkaloids, carbohydrates, polyphenolics, essential amino acids, and vitamins especially vitamin C [4]. Among these bioactive molecules, tannins had been reported for various therapeutic activities in cutaneous, pulmonary, duodenal, and colonic tumors [20, 21]. EOT had also been reported to potentiate the T cells and macrophages [42] and shown immunostimulatory effects in various human and animals models [41]. Keeping in view the diverse range of therapeutic and immunological activities of EO in different animal models, the present study reports the effects of EOT on humoral immune response in chickens and its protective efficacy against *Eimeria* infection (mixed species) in chickens.

In the current study, ICP-based elemental analysis was carried out to rule out the possibilities of heavy metal(s) in EO, as reported in some previous studies [43]. Heavy metals were reported to have adverse effects on the cells of innate immune system and caused inappropriate activation of the immune cells [44]. So, for safer use of EO, different minerals and heavy metals were detected in EO powder and all the minerals and metals detected were found in permissible intake levels, as recommended by World Health Organization [45].

For safety testing, a preliminary pilot project was conducted to find out the safe dose limit of tannins for use in actual experimental trial. Earlier, toxic effects of tannins in higher doses had been reported by Iqbal et al. [46] which caused adverse effects on the health. Based upon the findings of pilot project, the dose limit ≤ 1 gm/Kg of body weight was found to be safe because no adverse effects were noted in chickens administered with tannins at this dose rate, whereas chickens administered with higher doses showed abnormal physiological and behavioural signs.

In the current study, SRBCs were used as nonpathogenic T-cell dependant immunogens [47, 48] to demonstrate the effect of EOT on the humoral immune response in chickens. EOT exerted stimulatory effects on humoral immune responses in chickens. Oral administration of EOT resulted in higher total Igs, IgG, and IgM antibody titers against SRBCs on days 7 and 14 post primary and secondary injections of SRBCs, when compared with those administered with CTA and PBS administered control group. Results of the present study are contrary to the earlier findings of Marzo et al. [42] in which tannic acid was reported to reduce the levels of total Igs, IgG, and IgM in a dose-dependant manner. On the other hand, some previous studies also reported the stimulatory effects of tannins on the expression of cytokines [49] that might be correlated with higher antibody titers in tannins administered chickens. The immunosuppressive effect of tannic acid reported in previous studies might be due to its higher concentration which had toxic and detrimental effects on health [46, 50]. Further, difference in the source of tannic acid and extraction method used could also be the possible reasons of variation in results. Higher doses of tannic acid put the birds under stress which elevated the corticosterone levels in plasma that might be involved in the impairment of immune system [51]. Therefore, it may be speculated that dose of tannic acid administered to the birds is important to get the beneficial outcomes.

Nonsignificant effect of tannins was detected on the development of lymphoid organs in EOT-administered chickens as compared to control groups. Analogous findings have also been reported in similar studies [37, 52].

In challenge experiment, protective efficacy against coccidial infection was determined by the increase in daily body weight gains and oocysts per gram of droppings after challenge with *Eimeria* species. Higher daily weight gain and reduced oocyst shedding are considered to be important indicators of host's resistance to coccidian infection, although direct correlation between the two parameters was not recorded in the present study like previous studies [53, 54]. In case of coccidiosis, some medicinal foods and probiotics had been reported to provide protection against the infection by potentiating the specific immune responses, particularly the cellular and humoral, against *Eimeria *infection in chickens [33, 55].

In the current study, all the experimental groups administered with graded doses of EOT showed significantly lower (*P* < 0.05) OPG as compared to control. Among the experimental groups, the difference in OPG was statistically similar (*P* > 0.05) between the groups administered with CTA or EOT on a particular dose rate and day after infection. The lower OPG might be due to the lethal effect of tannins on the growth and proliferation of parasite as reported in case of coccidial [56] and most of the helminth infections [57]. Further, Min and Hart [58] also reported that tannins may form complexes with nutrients and inhibit their availability to the parasite for normal growth, development, and motility and thus decrease the metabolism of parasite directly through inhibition of oxidative phosphorylation or electron transport.

The results of daily weight gains showed significantly higher (*P* < 0.05) weight gains in EOT and CTA administered chickens as compared to PBS administered control group, whereas difference between the groups administered either with EOT or CTA was statistically similar. Further, chickens administered with EOT were active with normal feed and water intake, normal behavior, and no/least abnormal signs/symptoms. On the other hand, chickens in control groups were dull and depressed with ruffled feather and took less feed and water that might be due to certain modifications in gut homeostasis, which altered the bird's metabolism that led to less feed intake and decreased weight gains [59–61].

Results of percent protection revealed maximum protection (53.3–60%) in EOT administered groups followed by those administered with CTA (40–46.67%) and control group (26.62%). The protection in control group might be due to the self-limiting nature of coccidial parasites in birds during the course of infection [62].

In challenge experiment, results showed higher percent protection against caecal and intestinal lesions in EOT-administered chickens followed by those of CTA and negative control groups, respectively. Decreased damage to the caecal mucosa in EOT administered chickens suggested the involvement of some immune effector components present in the tannins that might inhibit the development of the parasites life cycle in the host [63]. During coccidial infection, the cytokine metabolite environment, produced within the microenvironment of the bird's intestine, may lead to physiological alterations including vasodilation which caused increased hemorrhagic lesions in severely infected negative control chickens [64]. Further, McCann et al. [65] also reported a reduction in detrimental impact of coccidiosis on the intestinal tract in the form of lower lesion scores in chickens fed on tannin-supplemented diets.

In the present study, a significantly higher humoral response (*P* < 0.05) against *Eimeria* species was noted in chickens administered with tannins as compared to those of control group. Antibodies have been reported to have an important role in conferring the protective immunity against *Eimeria* species in infected chickens [66]. Further, antibodies can efficiently inhibit the development of *Eimeria* in the intestine [67]. A positive correlation between antibody titers and protection against coccidiosis has also been reported earlier [68, 69]. In some previous studies, antibodies have been shown to provide the partial protective passive immunity by hampering the growth, development, and multiplication of parasite [70, 71]. In this study, the therapeutic efficacy of tannins might be attributed to their stimulatory effects on the production of antibodies against experimentally induced *Eimeria* species and thus leading to higher weight gains and lower OPG.

In conclusion, the results of the present study showed that EOT may be a potential and valuable candidate to potentiate the humoral immune responses in chickens and can be used successfully as a protective agent against coccidiosis. Further, it can also be exploited as a low-cost alternative to allopathic drugs for the control of avian coccidiosis. Further studies are needed to elucidate the specific type, structure, and function of EOT and the mechanism(s) involved in such immunostimulatory and protective activities in chickens.

## Figures and Tables

**Table 1:** Inductively coupled plasma (ICP) based elemental analysis of *Emblica officinalis *dried powder.

Elements (symbols)	Detected wavelength (nm)	Detected values (mg/L)
Arsenic (As)	193.66	—
Barium (Ba)	233.527	0.009
Cadmium (Cd)	228.802	—
Cobalt (Co)	228.616	—
Copper (Cu)	327.393	—
Iron (Fe)	238.204	0.414
Potassium (K)	766.490	172.4
Lithium (Li)	670.784	—
Magnesium (Mg)	285.213	8.724
Manganese (Mn)	257.610	0.135
Nickel (Ni)	231.604	0.005
Lead (Pb)	220.353	—
Zinc (Zn)	206.200	0.062
Strontium (Sr)	407.771	—

All detected values are under permissible intake level as recommended by WHO (Anonymous, 1999).

**Table 2:** Antibody titers (geomean titres) to sheep red blood cells in experimental and control groups.

Total Immunoglobulins				
Group	Day 7 PPI	Day 14 PPI	Day 7 PSI	Day 14 PSI
A_1_	24.25	27.86	32.00	42.22
B_1_	21.11	27.86	32.00	36.76
A_2_	36.76	42.22	55.72	64.00
B_2_	27.86	32.00	48.50	55.72
A_3_	32.00	36.76	64.00	64.00
B_3_	32.00	32.00	42.22	53.82
C	18.38	21.11	24.25	26.91

Immunoglobulin M				
A_1_	18.19	11.86	16.00	10.22
B_1_	13.11	11.86	13.62	8.9
A_2_	26.20	17.97	23.72	15.50
B_2_	18.67	13.62	24.25	13.50
A_3_	22.81	15.65	27.24	8.28
B_3_	24.00	13.62	17.97	11.59
C	13.79	8.99	10.32	7.88

Immunoglobulin G				
A_1_	6.06	16.00	16.00	32.00
B_1_	8.00	16.00	18.38	27.86
A_2_	10.56	24.25	32.00	48.50
B_2_	9.19	18.38	24.25	42.22
A_3_	9.19	21.11	36.76	55.72
B_3_	8.00	18.38	24.25	42.23
C	4.59	12.13	13.93	19.03

A_1_:  *Emblica officinalis *derived tannins at 0.50 gm/Kg b.wt; B_1_: commercial tannins at 0.50 gm/Kg b.wt; A_2_:* Emblica officinalis *derived tannins at 0.75 gm/Kg b.wt; B_2_: commercial tannins at 0.75 gm/Kg b.wt; A_3_: *Emblica officinalis *derived tannins at 1.00 gm/Kg b.wt; B_3_: commercial tannins at 1.00 gm/Kg b.wt; C: PBS-administered negative control.

**Table 3:** Percent protection against mortality and intestinal, caecal lesions in experimental and control chickens.

Group	Protection (%)	Protection against lesions (%)	
		Intestine	Caeca
A_1_	53.33	23.25	20.00
B_1_	46.67	20.00	18.25
A_2_	60.00	25.00	21.75
B_2_	46.67	20.00	17.50
A_3_	53.33	21.75	30.00
B_3_	40.00	15.00	15.00
C	26.67	13.25	10.00

A_1_: *Emblica officinalis *derived tannins at 0.50 gm/Kg b.wt; B_1_: commercial tannins at 0.50 gm/Kg b.wt; A_2_: *Emblica officinalis *derived tannins at 0.75 gm/Kg b.wt; B_2_: commercial tannins at 0.75 gm/Kg b.wt; A_3_: *Emblica officinalis *derived tannins at 1.00 gm/Kg b.wt; B_3_: commercial tannins at 1.00 gm/Kg b.wt; C: PBS-administered negative control.
